# Increasing productivity by using smart gas for optimal management of the gas lift process in a cluster of wells

**DOI:** 10.1038/s41598-024-63506-w

**Published:** 2024-07-05

**Authors:** Jalal Abu-Bakri, Arezou Jafari, Hamed Namdar, Goodarz Ahmadi

**Affiliations:** 1 Petroleum Engineering Department, Faculty of Chemical Engineering, Tarbiat Modares University, Tehran, Iran; 2 Department of Mechanical and Aerospace Engineering, Clarkson University, Potsdam, NY USA

**Keywords:** Artificial gas lift, Smart gas, Optimization, Greenhouse gases, Oil production, Geodynamics, Petrology, Environmental impact

## Abstract

In the face of the escalating global energy demand, the challenge lies in enhancing the extraction of oil from low-pressure underground reservoirs. The conventional artificial gas lift method is constrained by the limited availability of high-pressure gas for injection, which is essential for reducing hydrostatic bottom hole pressure and facilitating fluid transfer to the surface. This study proposes a novel ‘smart gas’ concept, which involves injecting a gas mixture with an optimized fraction of CO_2_ and N_2_ into each well. The research introduces a dual optimization strategy that not only determines the optimal gas composition but also allocates the limited available gas among wells to achieve multiple objectives. An extensive optimization process was conducted to identify the optimal gas injection rate for each well, considering the limited gas supply. The study examined the impact of reducing available gas from 20 to 10 MMSCFD and the implications of water production restrictions on oil recovery. The introduction of smart gas resulted in a 3.1% increase in overall oil production compared to using natural gas. The optimization of smart gas allocation proved effective in mitigating the decline in oil production, with a 25% reduction in gas supply leading to only a 10% decrease in oil output, and a 33% reduction resulting in a 26.8% decrease. The study demonstrates that the smart gas approach can significantly enhance oil production efficiency in low-pressure reservoirs, even with a substantial reduction in gas supply. It also shows that imposing water production limits has a minimal impact on oil production, highlighting the potential of smart gas in achieving environmentally sustainable oil extraction. Furthermore, the implementation of the smart gas approach aligns with global environmental goals by potentially reducing greenhouse gas emissions, thereby contributing to the broader objective of environmental sustainability in the energy sector.

## Introduction

The need for energy increases proportionally to the economy’s and population’s growths^1–3^. As the oil reservoir pressure decreases, its natural production reduces, and less oil is delivered to the surface^4,5^. When the oil reservoir pressure is less than that required for natural production, it is necessary to employ one of the artificial lift techniques to bring an acceptable amount of oil to the surface^6–8^. The petroleum industry has advanced to a new level in extensively using cognitive computing and digitalization. Also, time-consuming tasks that require accuracy are benefiting from using artificial intelligence^9,10^.

For extracting oil from old wells, various approaches are utilized. The most prevalent and cost-effective approach in the oil industry is artificial gas lift^11–17^. In this method, high-pressure gas is injected into the tubing, decreasing the fluid density in the well. This leads to a decrease in the bottom hole pressure and an increase in flow rate^18–22^. However, high-pressure gas also has huge risks to nearby wells and surface facilities in case of leakage during the gas lift process^23^. The optimum design of gas-lift operation significantly enhances production and avoids economic losses. To fully account for all reservoir, well, and facility restrictions, it is crucial to treat the gas-lift model as an optimization process using an integrated modelingapproach^24^.

Significant modelings of oil and gas fields for flow management, production, and stability were performed. These models use the mass balance of the various fluid phases as the basis for their optimization^25–27^. Generally, the optimal amount of gas injection plays an important role in the gas lifting process. The gas Lift performance curve (GLPC) is a curve that shows the rate of oil production versus the rate of gas injection. The GLPC curves typically have a concave shape, which shows a point of maximum oil production. Therefore, if less gas is injected during the gas lifting process, the oil production rate decreases; on the other hand, if the amount of gas injected is excessive, the frictional pressure gradient causes the oil production rate to decrease and the operation costs to rise. Each well has a peak performance rate where it produces the most oil under optimal conditions^28,29^. In an ideal case, if there is no limitation in the gas resources and facility, the maximum production rate can be achieved by injecting sufficient gas into the well^30^.

For obtaining the highest possible production in an old field with several wells, generally, the optimization of the gas allocation is needed because, in most circumstances, the compressing unit’s capacity and the available gas are restricted. Therefore, optimizing gas allocation for the gas lifting process for a collection of wells must be performed^7,30,31^. Gas allocation optimization problems generally include two steps: 1- gas lift performance curves (GLPC) and 2- optimization of gas allocation among wells^32,33^. The performance curve is the first phase in the investigation of the impact of gas injection in the gas lift process^34,35^. The GLPC significantly impacts the efficient allocation of gas. This is due to the fact that the amount of gas injection has an optimal value from the field development and operational and economic standpoint^30,32^.

**Figure 1:**
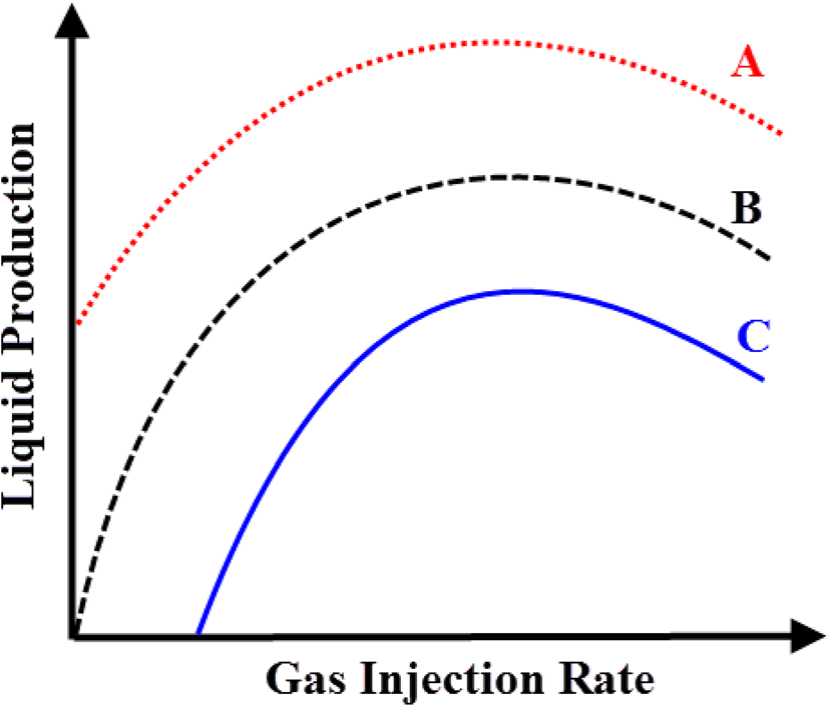
Types of gas lift performance curve.

In terms of the performance curve, all gas lift wells can be divided into three categories, as shown schematically in Fig. 1. Curve A represents a well with a natural potential to produce, and by performing a gas lifting process, its production increases. Curve B represents a well with no natural production, and oil can be extracted only by performing gas lifting operations with a small amount of gas. A well that requires at least a minimum quantity of gas injection in order to begin producing is shown by Curve C^29,30^.

The GLPC analysis shows that the well’s production increases by gas injection. In addition, as more gas is injected, so does the production until the process reaches the maximum production point. Then, as the amount of gas injected rises, the well production declines, and costs rise^29,30^. Therefore, reaching the optimal point in each well’s gas lift performance curve is important^36^.

Comparative studies of nonlinear and piecewise linear optimation, including the intrinsic nonlinearity of the gas lift performance curve, were reported by^37^. They showed that it was vital to maximize the amount of gas allocated to each well, and the traditional optimization methods had significant challenges in handling the network of wells with a large number of constraints. Using an appropriate optimization technique would increase oil production^28^. Due to the limited gas supply, it is essential to maximize resource efficiency and control the gas injection rate into each well to increase production^38^. Also, it is better to optimize the whole field^39^. Case studies have demonstrated that the closed-loop optimizer has proven effective in preserving the compressor station’s output pressure and improving gas lift networks in the event of a compressor failure^40^.

Usually, in the gas lift process, natural gas is used as injected gas^41^. Gas lift by natural gas did not begin on a large scale until the 1920s. The initial operation of the gas lift was carried out using air as an injection gas. In large oil fields such as Goose Creek and Spindle Top, gas lift was carried out by air^42^. Air has disadvantages when used in gas lifting; when mixed with the well’s fluids, the oxygen in the air causes corrosion, deposition, and the possibility of combustion. Nitrogen and carbon dioxide are other options for the gas lifting process instead of natural gas. The advantages of using nitrogen gas are its lightness, neutrality, low cost, and anti-corrosion^43^. Also, carbon dioxide gas has been used for gas lifting in some wells; however, it can cause corrosion in equipment ^42^. However, the use of these gases requires additional costs for purification.

In this study, flue gas is used as the basis of the study to save the cost of purification and take advantage of carbon dioxide and nitrogen gas simultaneously. In addition, by modifying the composition of flue gas and optimizing it for each well, a smart gas is developed for injection in the process of gas lift so that, in addition to optimizing gas allocation, the maximum amount of oil can be extracted from the reservoir and the water cut can be minimized. In addition to environmental advantages (by removing flue gas from the environment), using this gas has economic advantages because natural gas, usually used in gas lift processes, can be provided to the customer in this case. This advantage is more important in the cold months when the demand for natural gas consumption increases. The prevailing methods for enhancing oil recovery from low-pressure reservoirs are increasingly inadequate due to the rising global energy demand and the limitations of existing technologies. The scarcity of high-pressure gas for artificial lift systems poses a significant bottleneck, often leading to suboptimal recovery rates and environmental concerns. Despite numerous advancements, the current research landscape is grappling with the challenge of optimizing resource allocation to maximize oil extraction efficiency. The conventional reliance on natural gas for injection is proving unsustainable, necessitating the exploration of alternative solutions that can operate effectively within the constraints of limited resources. This paper introduces the innovative ‘smart gas’ concept, a transformative approach that leverages an optimized mixture of CO_2_ and N_2_ to overcome the limitations of traditional gas lift methods. By employing a dual optimization strategy, this research not only pioneers a novel gas composition but also presents a pragmatic solution for the judicious allocation of scarce gas resources across multiple wells, thereby setting a new precedent in the field of sustainable oil extraction.

The presented results showed a significant increase in overall oil production with the use of smart gas. The total production increased by 3.1% when smart gas was used instead of natural gas for a 20 MMSCFD available gas injection, also, by employing optimization, the total oil production was only reduced by 10% and 26.8%, respectively, when the amount of available gas was reduced from 20 to 15 MMSCFD (25% drop) and from 15 to 10 MMSCFD (33% drop).

## Methodology

**Figure 2:**
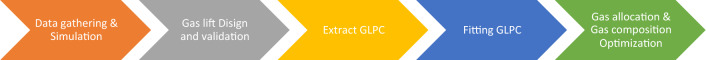
the procedures used in this study’s methodology.

First, information on 10 wells from an Iranian Offshore oil reservoir, including all necessary data, was collected, and the wells were modeled by PROSPER from the IPM software package. After validating the modeled wells, gas lift design was performed with 12 different combinations of carbon dioxide (CO_2_) and nitrogen (N_2_) as injection gas, and all GLPC data were extracted. Oil and water flow rates are evaluated versus the gas injection rate and composition by fitting the extracted GLPC curves with the help of models in the Design Expert software. The fitted models are used to optimize gas allocation among wells for finding the optimal composition of flue gas (smart gas) with the help of the Generalized Reduced Gradient (GRG) optimization algorithm. In the following sections, different scenarios are investigated by applying different restrictions. The study method is briefly outlined in Fig. 2.

### Well simulation

The gas lift design is underpinned by a detailed well simulation that is both comprehensive and precise model by production engineering software(Prosper). This simulation is integral to our understanding of the well’s behavior under various operational scenarios and is the foundation upon which we base our design decisions.

-Modeling process

1. Data Integration: The simulation begins with an exhaustive integration of all pertinent data, which forms the backbone of the well model. This data includes, but is not limited to, well trajectories, completion details, reservoir characteristics, and historical performance metrics.
2. Systematic Analysis: A systematic analysis using nodal analysis techniques is conducted to harmonize the VLP and IPR curves. The analysis is iterative, refining the model with each iteration to ensure it mirrors the real-world performance of the well.

-Modeling method

1. Graphical Method Refinement: The graphical method is refined to provide a clear visual representation of the pressure dynamics within the well. This involves a meticulous plotting of pressure gradients and careful consideration of valve pressure drops to ascertain the most effective placement of gas lift valves.
2. Predictive Accuracy: The model’s predictive accuracy is paramount. Adjustments are made to the simulation parameters to ensure they are representative of the actual reservoir conditions. This enhances the model’s ability to forecast the well’s response to different artificial lift techniques with a high degree of confidence. Simulation approach is meticulously crafted to align with the research objectives, emphasizing the innovative modeling techniques and the insights they yield.

### Reservoir, well, and PVT data

**Figure 3:**
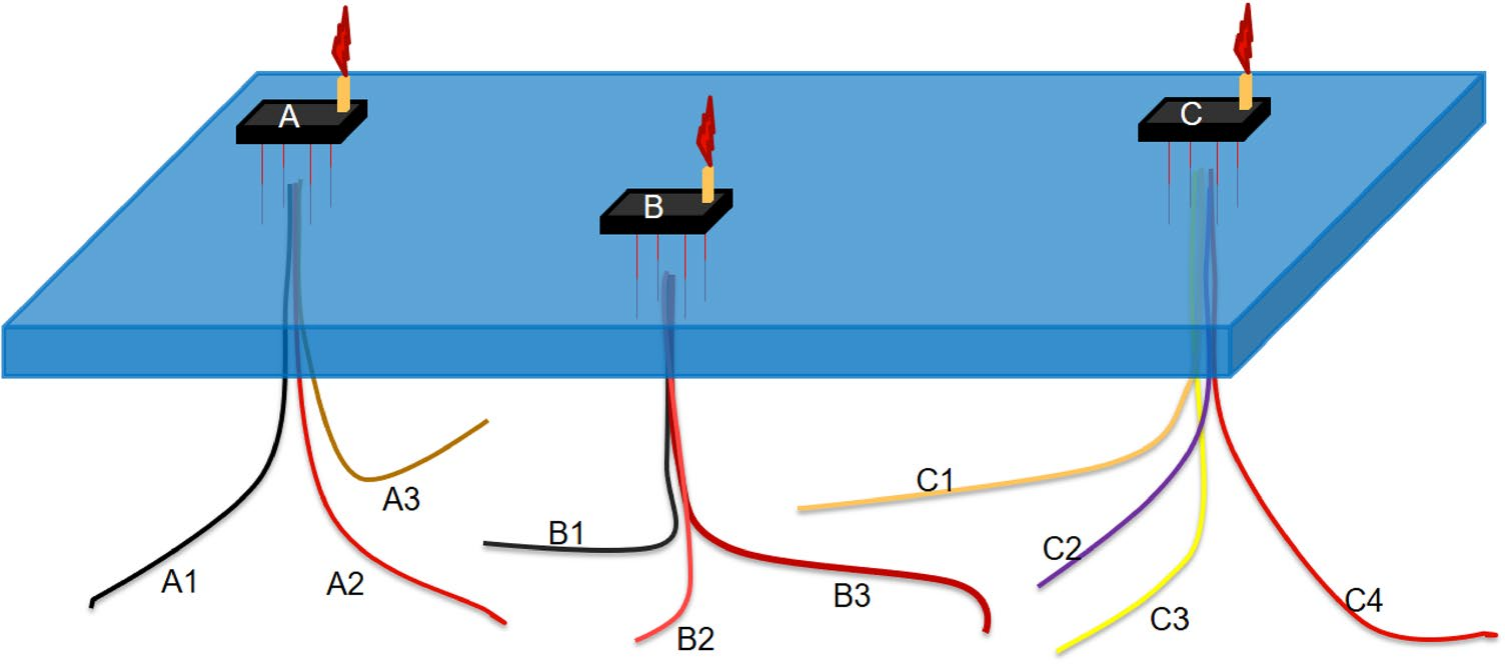
Schematic of candidate well.

**Figure 4:**
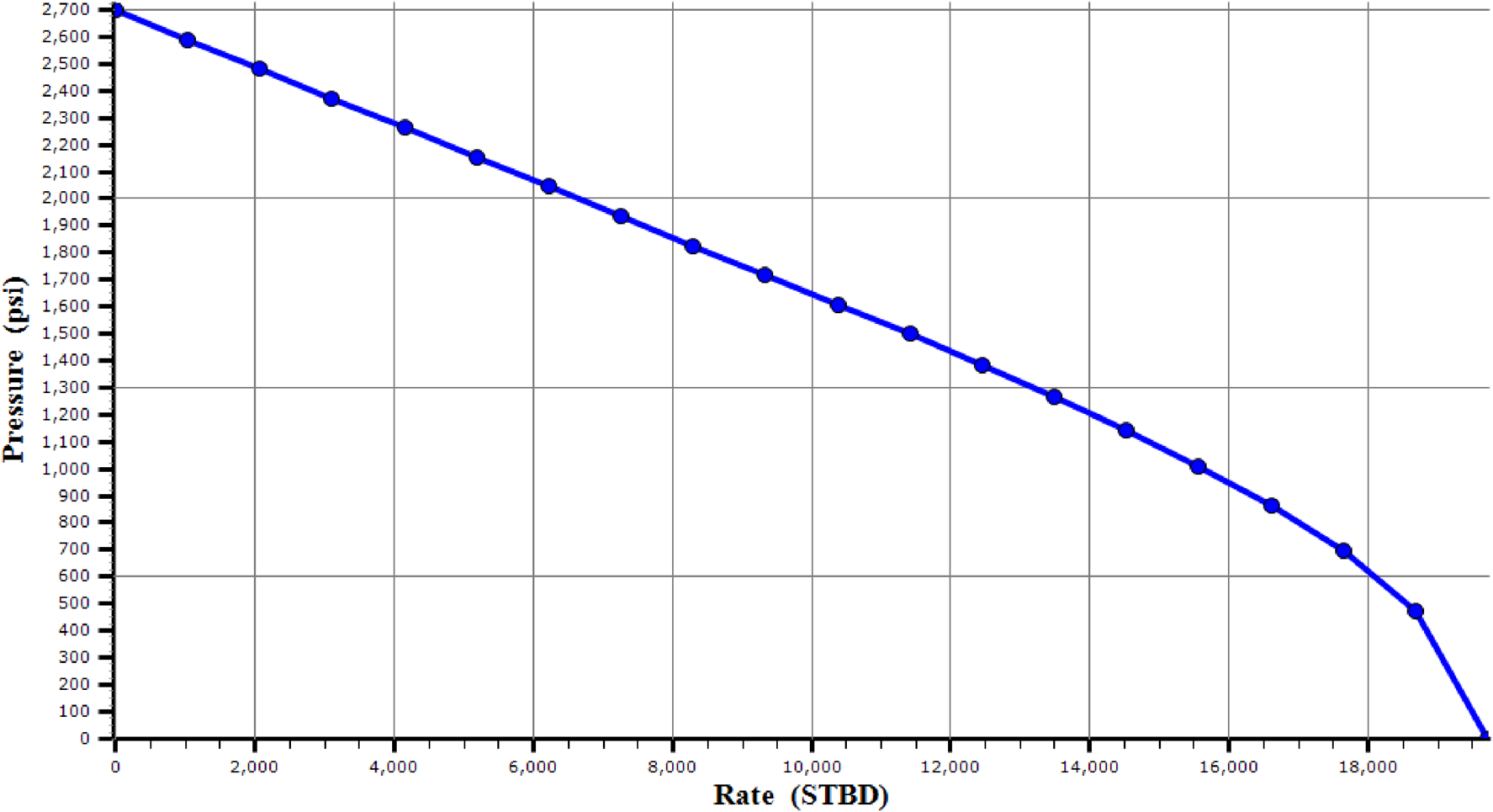
IPR model of a typical well.

Present study focuses on an offshore reservoir in the Persian Gulf, equipped with 10 production wells distributed across three platforms (Fig. 3). Over 43 years of production, the reservoir pressure has declined, necessitating the implementation of artificial lifting techniques to maintain oil production. The reservoir data is summarized in Table 1, while the Inflow Performance Relationship (IPR) for a typical well is depicted in Fig. 4.

**Table 1:** Initial characteristics of the reservoir for simulation.

Reservoir pressure (Psi)	2700
Reservoir temperature (F)	178
WCT%	50
Total GOR (SCF/STB)	400
PI (STB/Psi*Day)	9.5
AOF (STB/Day)	19,713

The reservoir exhibits a Total Gas Oil Ratio (GOR) of 400 SCF/STB, a pressure of 2700 psi, a temperature of 178°F, and a water cut of 50%. The productivity index for the candidate well is 9.5 STBD/psi, indicating the well’s ability to produce fluid per unit pressure drop. All wells were drilled horizontally to maximize exposure to the reservoir. The PI model, the simplest form of the reservoir model, is employed to define the IPR based on the available data. This model is instrumental in understanding the well’s performance by relating the production rate to the flowing bottom-hole pressure.

**Figure 5:**
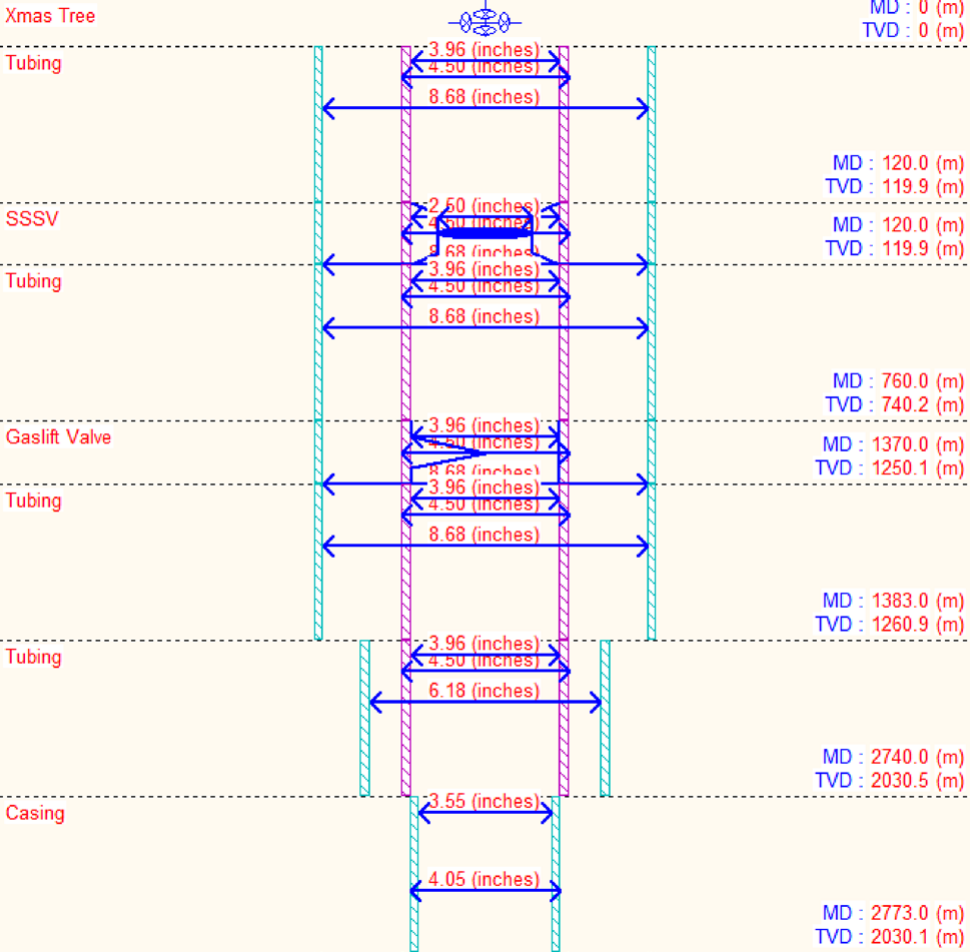
Downhole completion summary with depth.

The production casing has an outside diameter of 9 5/8 inches, while the tubing’s inner diameter measures 3.958 inches. A gas lift system is integrated within the well to assist in lifting the oil to the surface. Figure 5 illustrates the gas lift completion’s downhole summary, including the depth at which the gas is injected.

To evaluate the reservoir fluid behavior and ascertain the pressure drop across the well, detailed fluid characteristic information is essential. The PVT data parameters—solution GOR, water salinity, API gravity, and gas gravity—are pivotal in elucidating the thermodynamic behavior of the reservoir fluid. The black oil correlations are calibrated against laboratory-measured PVT data to ensure accuracy.

For calculations, we utilize Beggs’ correlation to determine oil viscosity and Glaso’s correlation for estimating the GOR, oil Formation Volume Factor (FVF), and Bubble Point Pressure (BPP). The total heat transfer coefficient is estimated at 8.2 BTU/h/ft2/°F, which, combined with the formation temperature of 178°F at a depth of 2922 m, allows us to calculate the geothermal gradient. The heat capacities for oil, gas, and water are 0.53, 0.51, and 1 BTU/lb/°F, respectively. In the Table 2 details the Fluid PVT Properties of the reservoir, providing essential data for modeling fluid behavior and well performance. The Solution GOR of 272 SCF/STB indicates the volume of gas per barrel of oil, while an API gravity of 25 reflects the oil’s relative density. A Gas Gravity of 0.7 and a Water Salinity of 210,000 ppm. The Bubble Point Pressure and Temperature, at 1345 psi and 176°F respectively, are critical for understanding the phase behavior of the reservoir fluids under changing conditions.

**Table 2:** Fluid PVT properties.

Solution GOR	SCF/STB 272	
OIL gravity	API 25	
Gas gravity	0.7	
Water salinity	ppm 210,000	
Bubble point	Pressure (Psi)	Temperature (F)
	1345	176

### Gas lift design

The main idea behind gas lift in oil wells is to reduce hydrostatic pressure loss in the tubing by lowering the density of the produced fluid. For designing the gas lift system, the key parameters must be evaluated. According to the available data, the injection pressure and total available gas are 1500 psi and 20 MMscf/day, respectively. Each well’s gas injection rate is determined based on the optimization method and operational limits. Also, wellhead pressure limitation was considered based on the sea-line pressure. Accordingly, it should be at least 1.8–2 times the sea-line pressure, which in this study is 500 psi.

### Gas lift performance curve

**Figure 6:**
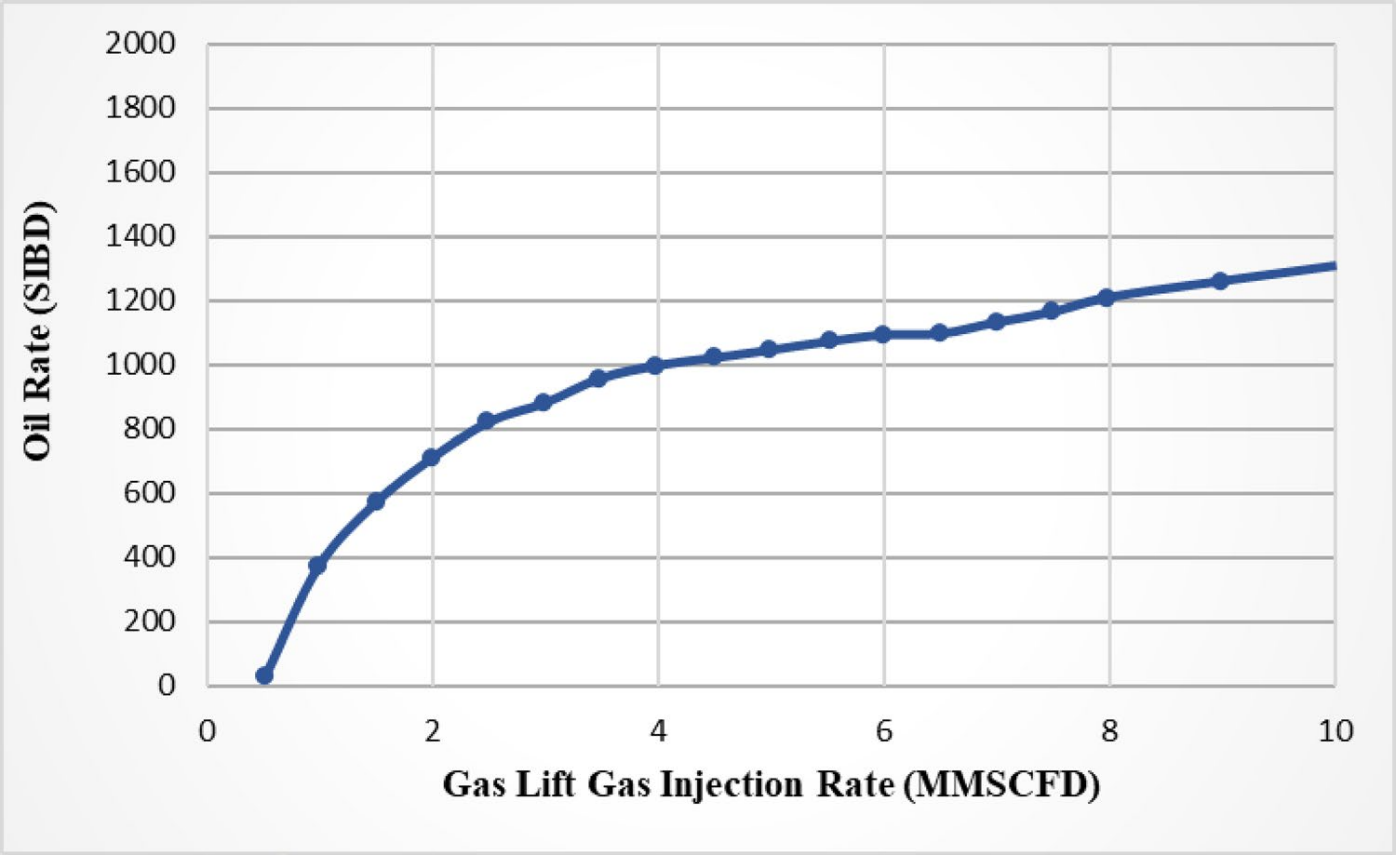
Gas Lift Performance curve of a typical well (well 1).

The influence of gas injection in the gas lifting design is first modeled using GLPC. The volume of gas injected into wells can be optimized by using an accurate GLPC. According to sea line pressure, the software generated the GLPC for all wells with a wellhead pressure of 500 psi, and due to the forecast of the field development, the water cut was taken 30% above the current water cut of each well. For each of the ten selected wells, 13 combinations of carbon dioxide and nitrogen gas as injection gas, two GLPCs, which represent the oil and water production, are developed and studied. Figure 6 shows the GLPC for a typical well (well 1). In regarding the potential disparity between simulated and actual production, it is important to note that while simulations provide a valuable framework for predicting outcomes, they are inherently subject to certain limitations. The actual production may differ due to a multitude of factors, including but not limited to, reservoir heterogeneity, equipment performance variability, and operational conditions that are not fully captured in simulation environments. To mitigate these discrepancies and enhance the practical application of the GLPCs, the study incorporates a robust validation process. Historical production data is utilized to calibrate the simulation models, ensuring that they closely mirror field performance. Additionally, sensitivity analyses are performed to understand the impact of key parameters on production forecasts, providing operators with a range of potential.

### Gas lift performance curves fitting

For optimization, an accurate relationship of the amount of oil or water production (response) vs the amount of gas injection and composition (factors) for each well is necessary. Therefore, the GLPC is fitted with empirical correlations utilizing the experimental design approach and Design Expert software. In this study, three different parameters were defined with gas injection flow rate at 11 levels from 0.5 to 5.5 MMSCFD as a numerical variable, nitrogen composition percentage (100-carbon composition percentage) at 12 levels from 0 to 100% as a numerical variable, and 10 wells models from well 1 to 10 as a nominal variable. Using tables of analysis of variance (ANOVA) and considering flow rate as response. Table 3 shows the experimental factors.

**Table 3:** Experimental design factors.

	Level 1	Level 2	Level 3	Level 4	Level 5	Level 6	Level 7	Level 8	Level 9	Level 10	Level 11	Level 12
Well	Well 1	Well 2	Well 3	Well 4	Well 5	Well 6	Well 7	Well 8	Well 9	Well 10		
Gas injection rate (MMSCFD)	0.5	1	1.5	2	2.5	3	3.5	4	4.5	5	5.5	
N_2_ Com. %	100	90	86	80	70	60	50	40	30	20	10	0

### Nonlinear gradient reduction optimization

In this study, the Generalized Reduced Gradient (GRG) algorithm employed to optimize the objective function concerning oil production under nonlinear constraints. The GRG algorithm is a robust method for solving nonlinear programming problems that involve both equality and inequality constraints. The GRG algorithm extends the principles of the Wolfe algorithm, which is designed for modified linear constraints, to accommodate nonlinear objective functions and constraints. It operates by iteratively converting the constrained problem into an unconstrained one, using a combination of the gradient of the objective function and a pseudo-gradient derived from the equality constraints. The Optimization Process will be as bellow.

1. Variable Categorization: Initially, the variables are split into dependent and independent sets. In the context of Excel’s Solver, these are referred to as basic and non-basic variables. 2. Search Direction: The algorithm determines a search direction using the generalized reduced gradient, which is a blend of the objective function’s gradient and the pseudo-gradient from the constraints. 3. Iterative Refinement: Through an iterative process, the GRG algorithm refines the solution by making small moves in the determined search direction, ensuring that any active constraint remains precisely active. 4. Application in this Study: The nonlinear GRG algorithm applied and using equations derived from Design Expert software for oil production. The optimization aimed to maximize oil production, assuming a total available gas volume of 20 MMSCFD, composed of nitrogen and carbon dioxide. 5. Constraints: The optimization was subject to the constraint that water production should not exceed 17,000 STBD. Additionally, the composition of gas injection optimized to enhance oil recovery. By integrating the GRG algorithm into the Excel solver tool, it can be able to navigate the complex landscape of our objective function effectively, taking into account the nonlinear nature of our constraints and arriving at an optimal solution for the composition of flue gas (smart gas) injection.

## Results and discussion

In this section, a polynomial fitted model for oil production as function of the amount of injected gas, and the composition of the smart gas is developed and the result of the optimization of flue gas composition and optimal allocation of smart gas between wells is described. Finally, the importance of optimizing the composition and allocation of gas in reducing production loss and applying various restrictions on water production is stated. Due to the industrial nature of the research, different scenarios have been discussed, and in case of necessity, the procedure can be changed from one mode to another.

### Statistical analysis of experimental design results

In this section, the statistical analysis of the planned experimental design was investigated, and the fitted models were analyzed using the analysis of variance (ANOVA. Based on the ANOVA results, the best model was selected for the subsequent analysis. The results of variance analysis for different fitted models are shown in Table 4. The F-value represents the proportion of the variance in the error compared to the variance in the model and is utilized to compare the variance of the variable or model to the error variance. The significance level is increased in models with higher F-values, and the model is not significant when the F-value gets closer to 1^44^. Also, if the P value of the model is less than 0.05, the significance of the model is confirmed with a confidence level of 95%^45^. The correlation coefficient is represented by the R^2^ value, which ranges from zero to one and indicates stronger correlations when the values are higher^46^. According to the results in Table 4, all the fitted models are significant, but the 4th-degree fitted model is more accurate because it has higher F and R^2^ values and is used in the rest of the study.

**Table 4:** Result of analysis of variance.

Fitted models	*P*-value	F-value	R^2^
Cubic	< 0.0001	270.03	0.9999
Quartic	< 0.0001	113.28	0. 9999
Fifth	< 0.0001	44.76	0.9996
Sixth	< 0.0001	15.12	0.9983

### Fitted model

The statistical analysis results show that 4th-degree regression, which is the model provided by the software, could correctly express the relationship between oil production, the amount of injected gas, and the composition of the smart gas. For example, the relationship of oil production flow rate in terms of gas injection flow rate and gas composition for well 1 is given in Table 5, where Qo is the rate of oil production, Qg is the rate of gas injection, and N2_Comp is the nitrogen composition percent.

**Table 5:** Proposed model for oil production of well 1.

The proposed model for oil production of well 1	
Well 1	$$\begin{aligned} {\text{Q}}_{{\text{o}}} & = - {524}.{86} + {25}0{9}.{41} \times {\text{Q}}_{{\text{g}}} + {1654}.{9}0 \times {\text{N}}_{{{2}\_}} {\text{Comp}} + {16}.{21} \times {\text{Q}}_{{\text{g}}} \times {\text{N}}_{{{2}\_}} {\text{Comp}} \\ & \quad - {822}.{63} \times {\text{Q}}_{{\text{g}}}^{{2}} - {2738}.{7} \times {\text{N}}_{{{2}\_}} {\text{Comp}}^{{2}} - {24}.{15} \times {\text{Q}}_{{\text{g}}}^{{2}} \times {\text{N}}_{{{2}\_}} {\text{Comp}} \\ & \quad + {136}.{24} \times {\text{Q}}_{{\text{g}}} \times {\text{N}}_{{{2}\_}} {\text{Comp}}^{{2}} + {128}.{18} \times {\text{Q}}_{{\text{g}}}^{{3}} + {2339}.{81} \times {\text{N}}_{{{2}\_}} {\text{Comp}}^{{3}} \\ & \quad - {1}.{4}0 \times {\text{Q}}_{{\text{g}}}^{{2}} \times {\text{N}}_{{{2}\_}} {\text{Comp}}^{{2}} + 0.{14} \times {\text{Q}}_{{\text{g}}}^{{3}} \times {\text{N}}_{{{2}\_}} {\text{Comp}} - {23}.{18} \times {\text{Q}}_{{\text{g}}} \\ & \quad \times {\text{N}}_{{{2}\_}} {\text{Comp}}^{{3}} - {7}.{31} \times {\text{Q}}_{{\text{g}}}^{{4}} - {86}0.{73} \times {\text{N}}_{{{2}\_}} {\text{Comp}}^{{4}} \\ \end{aligned}$$

**Figure 7:**
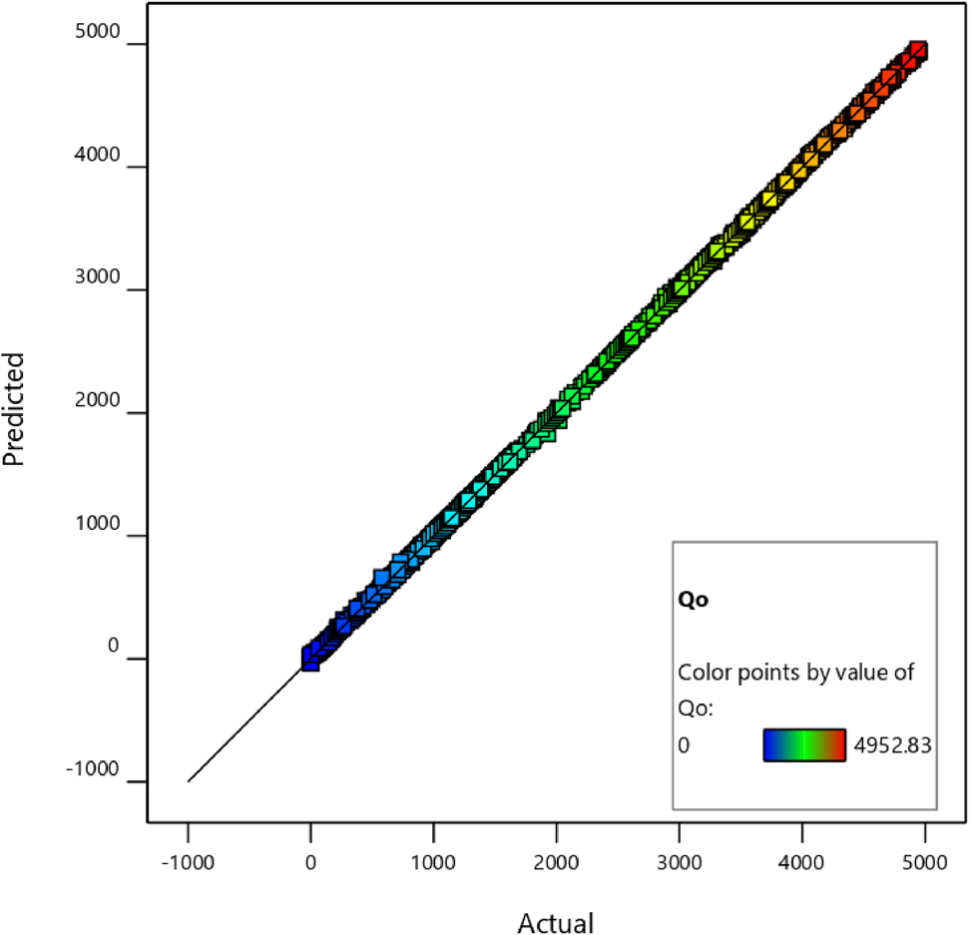
Predicted values vs. actual values.

Figure 7 presents a comparison between the predicted production value and the actual production data for Well 1. The graph illustrates how closely the model’s predictions align with the actual data. A straight line near the 45-degree angle indicates a strong correlation, signifying that the model’s predictions are accurate and reliable.

**Figure 8:**
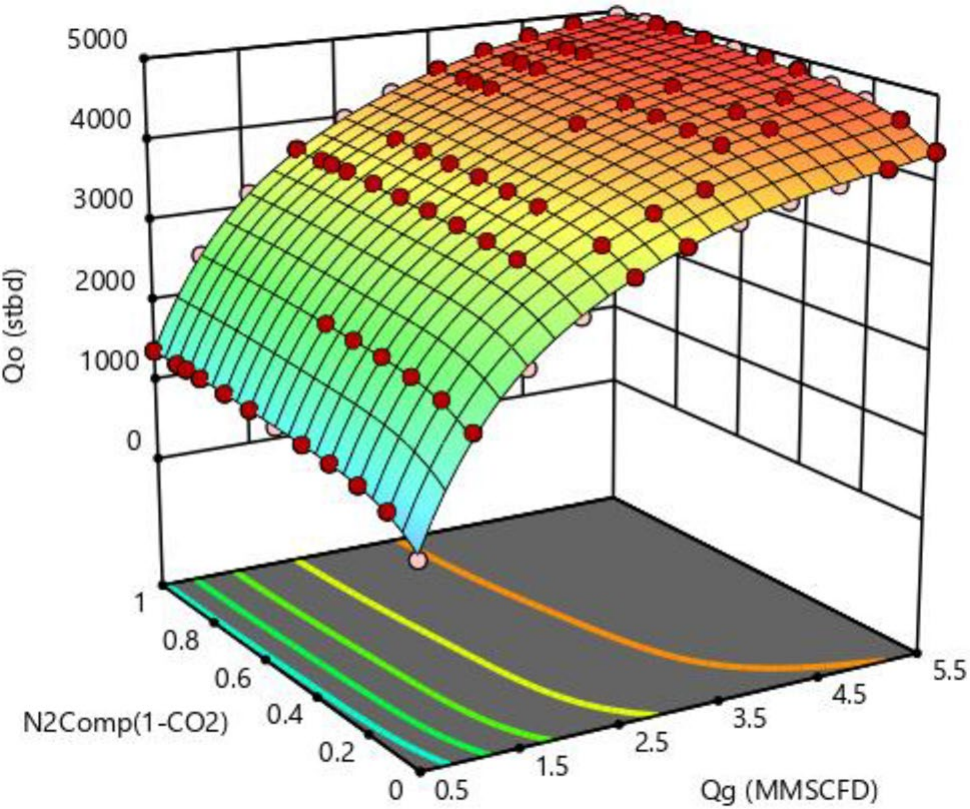
A three-parameter diagram of the design results of the oil production flow test for Well 1 according to the amount of injected gas and the composition of smart gas percentage.

The three-dimensional diagram of the effect of flow parameters and injection gas percentage composition on the oil production rate for well 1 is shown in Fig. 8. Since the injected gas is a mixture of nitrogen and carbon dioxide, this graph depicts how much oil production is obtained for Well 1 for different compositions of smart gas and injection amount. It is seen as the oil production increases as the gas flow increases. By increasing the percentage of carbon dioxide due to its high weight, the density of injected gas increases and requires more pressure to transfer oil to the surface; as a result, the amount of oil produced decreases. This importance is more apparent in percentages higher than 50% of carbon dioxide in injected gas. It should be noted that there is a three-dimensional diagram for oil and water production for each well.

### Optimization

In this section, based on the above analysis and considering all conditions, optimization of gas injection allocation and gas composition was performed for 20 MMSCFD available gas. To achieve maximum oil production despite the restriction of water production, which here is 17,000 STBD, the smart gas must be optimally allocated between the wells. Generally, the higher the productivity index and the lower the water cut of the well, the more gas is allocated to it. Table 6 shows the optimization of gas composition (smart gas) and allocation among wells. It clearly shows that wells 8 and 9, which have a higher productivity index and lower water percentage, have been optimized in such a way that more gas is allocated to them. On the contrary, wells 3 and 5 have less gas allocated to them due to lower productivity and higher water cuts.

**Table 6:** Optimizing gas composition (smart gas) and allocation among wells for 20 MMSCFD of available gas.

			Smart gas		
	Productivity index	Water cut	Q_g_ (MMSCFD)	Comp	
	STBD/psi	%		CO_2_	N_2_
Well 1	5	0.2	2.65	0	1.000
Well 2	7	8	1.30	0	1.000
Well 3	10	50	0.50	0	1.000
Well 4	9.5	50	1.22	0.039	0.961
Well 5	9	50	0.56	0	1.000
Well 6	9	40	1.48	0.03	0.970
Well 7	10	40	1.42	0.011	0.989
Well 8	20	5	3.83	0	1.000
Well 9	20	5	3.55	0	1.000
Well 10	9	5	3.49	0	1.000
Sum (MMSCFD)			20		
Total oil production (STBD)			21,605		

### Comparison of optimization of gas allocation between wells by smart gas, flue gas, pure gases, and natural gas

In the following sections, optimization results for the case of using natural gas, flue gas, and pure CO_2_ and N_2_ gases are compared. For this purpose, the different compositions of the gas containing pure CO_2_, pure N_2_, 86% N_2,_ and 14% CO_2,_ known as flue gas^47^ and natural gas, are considered. These cases are compared with smart gas and natural gas as injection gas in the gas lifting process. Table 7 shows that for all the different cases of gas injection, well number 8 has the largest share of gas injection. In the gas composition with higher percentages of CO_2_, higher gas volume was assigned to wells No. 3 and 5 due to low production. Also, Table 7 compares the total oil and water production by smart gas, flue gas, pure gases, and natural gas. The smart gas, which is the optimal flue gas composition, produces 21,605 STBD oil. The use of natural gas in the gas lifting process compared to smart gas has less production, about 672 STBD. As the percentage of carbon dioxide in the injected gas increases, its specific gravity increases. This reduces oil production in the artificial lifting process due to increasing fluid column density caused by increasing bottom hole pressure. For this reason, the lowest production is obtained by injecting pure CO_2_ (18,070 STBD), which has less production, about 3535 STBD relative to the smart gas. During the injection of pure N_2_, 21,504 STBD of oil is produced, which has less production, about 101 STBD relative to the smart gas. By increasing the percentage of carbon dioxide to 14% (flue gas), the oil production rate reached 21,386 STBD, which decreased by about 219 STBD of oil relative to the smart gas. Due to the fact that the initial composition of flue gas (86% N_2_, 14% CO_2_) is available and does not require purification, it is very affordable. Due to the appropriate composition of the injected gas, using smart gas results in the greatest quantity of oil production. The use of natural gas in the gas lifting process compared to smart gas, pure nitrogen, and flue gas leads to less production of about 672, 535, and 417 STBD, respectively.

**Table 7:** Optimization of gas allocation in the different combinations of injected gas compared to smart gas and natural gas.

	Pure CO_2_	Pure N_2_	Flue gas	Natural gas	Smart gas
	Q_g_ (MMSCFD)	Q_g_ (MMSCFD)	Q_g_ (MMSCFD)	Q_g_ (MMSCFD)	Q_g_ (MMSCFD)
Well 1	2.64	2.65	2.64	2.61	2.65
Well 2	0.99	1.31	1.29	1.27	1.30
Well 3	1.10	0.50	0.50	0.67	0.50
Well 4	1.39	1.23	1.24	1.21	1.22
Well 5	1.13	0.55	0.61	0.78	0.56
Well 6	1.65	1.48	1.48	1.50	1.48
Well 7	1.62	1.42	1.42	1.46	1.42
Well 8	3.37	3.82	3.82	3.74	3.83
Well 9	3.20	3.55	3.55	3.38	3.55
Well 10	2.93	3.49	3.46	3.37	3.49
Total injected gas (MMSCFD)	20	20	20	20	20
Total oil production (STBD)	18,070	21,505	21,386	20,933	21,605
Total water production (STBD)	15,205	17,000	17,000	17,000	17,000

If 20 MMSCFD of smart gas is used in the closed-loop gas lift process, approximately 107 MSCFD of CO_2_ will be removed from the environment. In the case of using flue gas as injection gas, the total amount of CO_2_ that will be removed is 2.8 MMSCFD, which is equivalent to 14% of the available gas. Finally, if pure carbon dioxide is used, CO_2_ is removed from the environment as much as the total volume of available gas, which here is 20 MMSCFD.

### Optimization in different amounts of available gas

This section discusses the impact of the total available gas injection on total oil production under different circumstances, including 10, 15, and 20 MMSCFD. The importance of this issue increases when natural gas is used as injected gas. Natural gas is a valued product that has significant economic and political implications. It’s crucial to conserve natural gas and switch to combustion gas. Gas allocation and optimization of smart gas combinations among wells were investigated, and the results are shown in Table 8.

**Table 8:** Gas allocation and smart gas composition for different available gas.

	10 MMSCFD			15 MMSCFD			20 MMSCFD		
	Q_g_ (MMSCFD)	Comp		Q_g_ (MMSCFD)	Comp		Q_g_ (MMSCFD)	Comp	
		CO_2_	N_2_		CO_2_	N_2_		CO_2_	N_2_
Well 1	1.31	0	1.000	1.77	0	1.000	2.65	0	1.000
Well 2	0.50	0	1.000	0.76	0	1.000	1.30	0	1.000
Well 3	0.50	0	1.000	0.74	0	1.000	0.50	0	1.000
Well 4	0.60	0.018	0.982	1.03	0.031	0.969	1.22	0.039	0.961
Well 5	0.50	0	1.000	0.87	0	1.000	0.56	0	1.000
Well 6	0.77	0	1.000	1.25	0.017	0.983	1.48	0.03	0.970
Well 7	0.86	0	1.000	1.25	0	1.000	1.42	0.011	0.989
Well 8	1.74	0	1.000	2.57	0	1.000	3.83	0	1.000
Well 9	1.71	0	1.000	2.42	0	1.000	3.55	0	1.000
Well 10	1.52	0.024	0.976	2.34	0	1.000	3.49	0	1.000
Total injected gas (MMSCFD)	10			15			20		
Total oil production (STBD)	15,805			19,407			21,605		
Total water production (STBD)	11,544			16,642			17,000		

Also, Table 8 shows the total production of the reservoir wells for different volumes of available injected gas. The results show that the reduction in available gas caused a drop in the total oil production. The important point in this table is that with the decrease in available gas from 20 to 15 MMSCFD (25% reduction), the total oil production decreases from 21,605 to 19,407 STBD, which is 10%. With a reduction in available gas from 20 to 10 MMSCFD (50% reduction), the total oil production decreases to 15,805, which is a drop of 26.8%. The results show that if the amount of available gas is reduced with proper management and optimization of gas composition and allocation, the decrease in oil production is minimized, which shows the importance of reservoir production management optimization. The result also shows that the 15 MMSCFD scenario can be replaced in the event of a gas shortage. The total water production for these two cases of available gas (10 and 15 MMSCFD) is 11,544 and 16,642, respectively, which are below the limit of water production.

### Optimization in different amounts of limited water production

Due to surface facilities limitation, the reservoir water production should be controlled. In this study and for 10 wells, according to the reservoir management and the surface facilities limitation, the total water production limit is 17,000 barrels. In order to consider the future conditions of well production, three scenarios have been considered containing 2000 barrels above and below the permitted water production limit and a state where there is no limit to the water production. Optimization of gas allocation and smart gas compositions among wells were investigated for different scenarios for the produced water, and the results are listed in Table 9. The most injected gas is assigned to well number 8. Wells No. 3 and 5 have the highest water production, and when the water production limit is applied, optimization is done so that less gas is allocated to these wells. In the case where there is no water production limit, the share of injected gas increases in wells with high water production, such as wells 3 and 5. Table 9 clearly shows that when there is no water production limitation, the injection rate into wells with high water production has grown significantly.

**Table 9:** Gas allocation and gas composition optimization for different limits of total water production.

	15,000 STBD of water			17,000 STBD of water			19,000 STBD of water			Without water production limit		
	Q_g_ (mmscfd)	Comp		Q_g_ (mmscfd)	Comp		Q_g_ (mmscfd)	Comp		Q_g_ (mmscfd)	Comp	
		CO_2_	N_2_		CO_2_	N_2_		CO_2_	N_2_		CO_2_	N_2_
Well 1	2.90	0	1.000	2.65	0	1.000	2.42	0	1.000	2.24	0	1.000
Well 2	1.41	0	1.000	1.30	0	1.000	1.19	0	1.000	1.09	0	1.000
Well 3	0.50	0.883	0.117	0.50	0	1.000	0.83	0.007	0.993	1.16	0.011	0.989
Well 4	0.94	0.038	0.962	1.22	0.039	0.961	1.32	0.038	0.962	1.41	0.033	0.967
Well 5	0.50	0.876	0.124	0.56	0	1.000	0.95	0.019	0.981	1.22	0.031	0.969
Well 6	1.14	0.038	0.962	1.48	0.03	0.970	1.59	0.022	0.978	1.69	0.013	0.987
Well 7	1.16	0.026	0.974	1.42	0.011	0.989	1.50	0.007	0.993	1.58	0	1.000
Well 8	4.01	0	1.000	3.83	0	1.000	3.59	0	1.000	3.40	0	1.000
Well 9	3.76	0	1.000	3.55	0	1.000	3.31	0	1.000	3.12	0	1.000
Well 10	3.68	0	1.000	3.49	0	1.000	3.30	0	1.000	3.10	0	1.000
Sum	20			20			20			20		

Table 10 shows the total produced oil and water for different limitations of total water production. Accordingly, if there is no restriction on water production, the total water and oil production would be 20,218 and 21,815 STBD, respectively. By applying water production restrictions, in the first scenario with water production restrictions up to 19,000 STBD, optimization has been done in such a way that even though 1200 STBD of water production has been reduced, only 58 STBD of oil production was reduced. In the other scenarios, with water production restrictions up to 17,000 and 15,000 STBD, oil production would decrease by approximately 152 and 703 STBD, respectively, despite the significant reduction in water production.

**Table 10:** The total produced oil and water for different limitations of total water production.

Water production limit (STBD)	15,000	17,000	19,000	Without water production limit
Total oil production (STBD)	21,054	21,605	21,757	21,815
Total oil production (STBD)	15,000	17,000	19,000	20,218

## Conclusions

Using smart gas instead of natural gas in the closed-loop gas lifting process provides significant economic advantages. It also somewhat reduces carbon dioxide in the environment (due to partially dissolving in oil). Wells modeling and sensitivity analysis provided the main design parameters, including gas injection pressure, flow rate, depth of injection valves, and wellhead pressure. Then, the GLPC curves for oil and water production were extracted for different combinations of nitrogen and carbon dioxide injection gases. Oil production optimization was performed for various constraints, including the available amount of gas, water production limitation, and optimal composition of injection gas (smart gas). Based on the findings of this study, the following conclusions were drawn:

1. The injection of smart gas, a strategic blend of flue gas components, yielded an oil production of 21,605 STBD, surpassing the outputs when natural gas, pure N2, pure CO2, and standard flue gas were used individually. Specifically, smart gas outperformed natural gas by 672 STBD, pure N2 by 101 STBD, pure CO2 by 3535 STBD, and standard flue gas by 219 STBD. This indicates that the synergistic effect of the combined gases in smart gas is more conducive to enhancing oil recovery than the separate injection of its constituents.
2. Without a water production limit, the total maximum amounts of oil and water produced were 21,815 and 20,218 STBD, respectively. Including water production constraints, in the first scenario with water production restrictions up to 19,000 STBD, optimization led to the reduction of 1,200 STBD water production, and only 58 STBD of oil production was reduced, which showed the high capability of smart gas in oil production management. In the other scenarios, with water production restricted to 17,000 and 15,000 STBD, oil production was decreased by approximately 152 and 703 STBD, respectively, despite the significant reduction in water production.
3. A reduction in the amount of available smart gas for a gas-lift injection resulted in a decrease in the oil production rate. Reducing the available injection smart gas from 20 to 15 MMSCFD (25% reduction) caused a decrease of 2200 STBD of total oil production while reducing the volume of smart gas from 20 to 10 MMSCFD (50% reduction led to a loss of 5800 STBD, which was a drop in total production by 10% and 26.8%, respectively.
4. The injected gas’s specific gravity increases as the fraction of carbon dioxide rises. The increased fluid column density enhanced the bottom hole pressure, and the oil production by the gas lift process was reduced. However, the magnitude of reduction can be reduced by suitable optimization.
5. 107 MSCF of CO_2_ would be removed from the environment if 20 MMSCF of smart gas was utilized in the closed-loop gas lift process for 10 wells. Using the flue gas as the injection gas, the total amount of CO_2_ that would be eliminated was 2.8 MMSCF (14% of the available gas). Finally, using pure carbon dioxide could remove as much CO2 from the environment as available gas, which is 20 MMSCF. For a field with 100 wells, the amount of CO2 gas used will be almost 10 times.
